# Development of the Digital Arthritis Index, a Novel Metric to Measure Disease Parameters in a Rat Model of Rheumatoid Arthritis

**DOI:** 10.3389/fphar.2017.00818

**Published:** 2017-11-14

**Authors:** Maria A. Lim, Brenton Louie, Daniel Ford, Kyle Heath, Paulyn Cha, Joe Betts-Lacroix, Pek Yee Lum, Timothy L. Robertson, Laura Schaevitz

**Affiliations:** ^1^Vium Inc., San Mateo, CA, United States; ^2^Capella Biosciences Inc., Palo Alto, CA, United States

**Keywords:** phenotypic screening, drug discovery screening, rheumatoid arthritis (RA), collagen induced arthritis, continuous monitoring platforms, rodent models of disease, 3rs strategies

## Abstract

Despite a broad spectrum of anti-arthritic drugs currently on the market, there is a constant demand to develop improved therapeutic agents. Efficient compound screening and rapid evaluation of treatment efficacy in animal models of rheumatoid arthritis (RA) can accelerate the development of clinical candidates. Compound screening by evaluation of disease phenotypes in animal models facilitates preclinical research by enhancing understanding of human pathophysiology; however, there is still a continuous need to improve methods for evaluating disease. Current clinical assessment methods are challenged by the subjective nature of scoring-based methods, time-consuming longitudinal experiments, and the requirement for better functional readouts with relevance to human disease. To address these needs, we developed a low-touch, digital platform for phenotyping preclinical rodent models of disease. As a proof-of-concept, we utilized the rat collagen-induced arthritis (CIA) model of RA and developed the Digital Arthritis Index (DAI), an objective and automated behavioral metric that does not require human-animal interaction during the measurement and calculation of disease parameters. The DAI detected the development of arthritis similar to standard *in vivo* methods, including ankle joint measurements and arthritis scores, as well as demonstrated a positive correlation to ankle joint histopathology. The DAI also determined responses to multiple standard-of-care (SOC) treatments and nine repurposed compounds predicted by the SMarTR^TM^ Engine to have varying degrees of impact on RA. The disease profiles generated by the DAI complemented those generated by standard methods. The DAI is a highly reproducible and automated approach that can be used in-conjunction with standard methods for detecting RA disease progression and conducting phenotypic drug screens.

## Introduction

Preclinical drug efficacy studies are an essential step in the drug discovery process. Despite promising preclinical results, a number of drugs that make it to the clinic fail to successfully translate to patients, thereby reducing the number of newly approved drugs (van der Staay et al., 2009; Jayasundara et al., 2012; Vincent et al., 2015). Target-based and phenotype screening platforms have been used in pharmaceutical and academic research to develop new therapeutics (Swinney and Anthony, 2011; Zheng et al., 2013; Vincent et al., 2015). While target-based screens focus on specific cellular or molecular targets, phenotypic screens focus on the modulation of disease-linked phenotypes (Vincent et al., 2015). In the past few years, there has been renewed interest in phenotype-based screens (Zheng et al., 2013).

Phenotypic screening in animal models of disease plays a crucial role in understanding pathogenic mechanisms and developing novel therapeutics. However, several challenges remain with using current clinical methods to evaluate disease in these animal models, including the subjective nature of semi-quantitative, scoring-based methods (Crabbe et al., 1999; Crawley, 2008; Mandillo et al., 2008; Scott et al., 2008; van der Staay et al., 2009; Zheng et al., 2013; Perrin, 2014; Sakic et al., 2015), time-consuming and labor-intensive longitudinal experiments, as well as the need for better functional readouts that represent all aspects of disease, thereby enabling greater translation to human diseases (Bendele, 2001; Roy et al., 2007; Asquith et al., 2009; Bevaart et al., 2010; Misharin et al., 2012). Therefore, there is a need for more consistent, automated, and clinically relevant methods to generate comparable and reproducible data for assessing disease phenotypes.

Rheumatoid Arthritis (RA) is a chronic, systemic disorder characterized by inflammation of joints and subsequent destruction of bone and cartilage (Sewell and Trentham, 1993; Firestein, 2003). Animal models of RA highlight many of the challenges in translational medicine. RA is a complex autoimmune disease arising from an interplay of genetic and environmental factors (Mcinnes and Schett, 2011). Patients typically present with mild joint stiffness and pain. As the disease progresses, these signs worsen, gradually affecting mobility, functionality, and ultimately quality of life (Firestein, 2003). Pathologically, RA is characterized by synovial inflammation, swelling, and autoantibody production, resulting in the destruction of cartilage and bone (Asquith et al., 2009; Mcinnes and Schett, 2011) in the small joints of the hands, wrists, and feet (Kahlenberg and Fox, 2011). Symptoms evolve over time and are thus monitored longitudinally in order to both capture disease progression and inform the appropriate course of treatment.

Various rodent models of immune-mediated arthritis are currently used in RA research (Bendele, 2001; Roy et al., 2007; Asquith et al., 2009; Misharin et al., 2012). Two of the most common experimental models of RA, with translation to the clinic, are adjuvant-induced arthritis (AIA) and collagen-induced arthritis (CIA) (Bendele, 2001; Caplazi et al., 2015). The CIA rodent model of RA is an invaluable model because of its simplicity, rapid disease onset, and reproducibility (Bendele, 2001; Bevaart et al., 2010). A number of currently approved anti-inflammatory or disease modifying anti-rheumatic drugs (DMARDs) have demonstrated positive effects in these models (Hegen et al., 2008). Many of the pathophysiological features of human RA are present in the CIA model, including cartilage destruction, inflammatory cell infiltration, production of rheumatoid factor, bone resorption and proliferation, obvious synovitis, and periarticular inflammation (Bendele, 2001; Asquith et al., 2009).

In rodent RA models, arthritic symptoms are measured daily either by manually measuring inflammation and swelling of the paws (Bendele, 2001; Brand et al., 2007) or using imaging modalities, such as magnetic resonance imaging (MRI) or radiography (Mountz et al., 2012). It is also common to obtain terminal histopathology in order to assess drug efficacy. In early stage drug discovery, trained technicians record visual scores of redness and swelling, as well as use calipers or a plethysmometer to manually measure the size of affected joints or the degree of paw swelling indexed by water displacement, respectively. To improve the consistency and reliability of measurements, the same technician is often responsible for collecting data across the entirety of the study. Manual joint measurements and clinical scores provide valuable information about the inflammatory response; however, these methods may not necessarily reflect structural damage and functionality, especially later in the disease (Sevilla et al., 2015; Song et al., 2015). In advanced stages of drug development, joint measurements and clinical scores are paired with *in vivo* imaging of arthritic joints, revealing valuable information about underlying structural changes. However, this method requires expensive equipment and, due to the time-consuming nature, a limited number of observations are available for assessing the efficacy of potential therapeutics. Furthermore, these measurements may not capture additional clinically relevant observations, such as loss of mobility, which dramatically affect quality of life in patients (Welsing et al., 2001; Pincus et al., 2004; Yazici et al., 2004; Bombardier et al., 2012).

We present a novel, automated, low-touch digital platform that can be used in-conjunction with standard methods to non-invasively assess disease and to evaluate the efficacy of drug candidates in well-established preclinical animal models. As a proof-of-concept, we validated our system in a CIA rodent model of RA. Using our digital platform, we created the Digital Arthritis Index (DAI) and validated the utility of this metric by demonstrating that it tracks standard methods of assessing arthritis across independent experiments and multiple standard-of-care (SOC) RA drugs with a high degree of reproducibility and predictive validity. We also screened several compounds approved for treating diseases, showed comparability between the DAI, standard measurements, and histopathology, then demonstrated how these methods complement one another in generating compound profiles. Our results demonstrate the power of an automated and objective digital platform with utility not only in RA drug discovery, but also with potential broad applications for phenotyping and screening compounds in other animal models of disease.

## Materials and Methods

### Animals

Experiments were conducted in Vium’s AAALAC-accredited Digital Vivarium in accordance with the National Institutes of Health (NIH) Guide for the Care and Use of Laboratory Animals and were approved by Vium’s Institutional Animal Care and Use Committee. Rats were singly housed in Vium Digital Smart Houses and were maintained on a 12-h light-dark cycle (06:00–18:00 PDT) with uniform, cage-level illumination and *ad libitum* access to food (Pico Rodent Diet 20 EXT IRR, LabDiet, Inc.) and water. Temperature, humidity, and airflow were monitored continuously for each cage.

### Collagen-Induced Arthritis Rat Model

**FIGURE 1:**
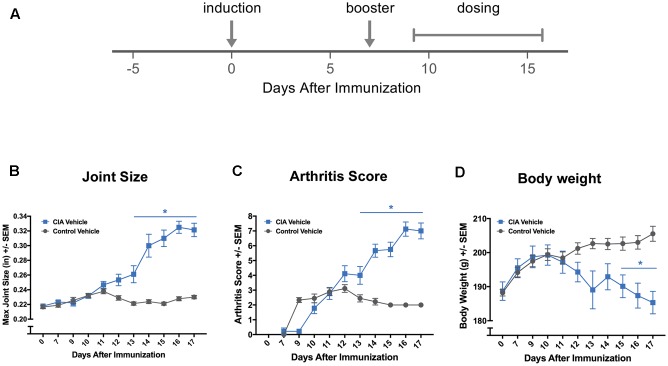
Standard methods confirm effective disease induction. **(A)** Experimental timeline of standard collagen-induced arthritis (CIA) study showing induction of arthritis with an injection of collagen + Incomplete Freund’s Adjuvant (IFA) and the booster injection (arrows), as well as the vehicle dosing schedule (brackets). Conventional measurements that are manually collected in a CIA study, such as joint measurements **(B)**, clinical arthritis scores **(C)**, and body weight **(D)** confirmed effective disease induction. ^∗^*P* ≤ 0.05 vs. Control Vehicle. Error bars are ±SEM. *n* = 9/group.

Female Lewis rats, weighing between 175 and 200 g (Charles River Laboratories; Hollister, CA, United States), were used in these studies. Subjects were randomized into study groups, such that all groups had similar average baseline body weights and activity profiles prior to induction. We employed the CIA model, a well-established and validated rodent rheumatology model (Bendele, 2001; Bevaart et al., 2010; Bolon et al., 2011). Briefly, to induce arthritis, rats were anesthetized under isoflurane and administered intradermal injections of 2 mg/mL of porcine type II collagen (Chondrex; Redmond, WA, United States) in Incomplete Freund’s Adjuvant (IFA) (Sigma; St. Louis, MO, United States) at two sites on the back of the animal. A booster was given 7 days post-induction. Control animals were injected with IFA only (**Figure 1A**).

### Prophylactic Treatment with Standard-of-Care Drugs

Rats were treated orally (PO) with either one of three clinical SOC drugs or vehicle beginning 9 days after the induction procedure, but before the onset of disease. Examined drugs included methotrexate (0.075 mg/kg, PO, qd) (Sigma; St. Louis, MO, United States), etanercept (Enbrel^®^, 10mg/kg, subcutaneously [SC], q3w) (Amgen; Thousand Oaks, CA, United States), dexamethasone (0.075 mg/kg, PO, qd) (MWI; Grand Prairie, TX, United States), or vehicle (PO, qd). All experimenters were blinded to treatment groups during the study.

### Treatment with Repurposed Compounds

Additional rats were treated with nine repurposed compounds identified using the SMarTR^TM^ computational drug discovery engine (Capella Biosciences Inc., Palo Alto, CA, United States). The SMarTR^TM^ engine is a highly automated and scalable computational platform tailored to understand disease heterogeneity through pathways and markers, as well as to identify potentially efficacious compounds relevant to a given disease. Nine compounds were predicted to have varying efficacies for the treatment of RA. Dose and route of administration for these compounds were selected based on previous pharmacokinetics and efficacy studies in other therapeutic areas. The vehicle or compounds (administered at a low, medium, or high dose) were given daily beginning 9 days after the induction procedure, but before the onset of disease. Compounds were administered either by PO or intraperitoneal (IP) routes and formulated in either 10% ethanol for PO dosing or 25% dimethyl sulfoxide (DMSO) for IP dosing. Phenanthridinone (Santa Cruz Biotechnology, Dallas, TX, United States) was administered by IP at 0.3, 10, and 30 mg/kg. Ouabain octahydrate (Sigma, St. Louis, MO, United States) was administered by IP at 0.2, 1.0, and 2.0 mg/kg. Beclomethasone (Sigma, St. Louis, MO, United States) was administered by PO at 0.16, 1.6, and 8.0 mg/kg. Celecoxib (Sigma, St. Louis, MO, United States) was administered by PO at 10, 20, and 38 mg/kg. To counter-balance for any effects of dosing route, animals administered PO doses of compounds also received IP injections of vehicle, while animals administered IP injections of compounds also received PO gavage. All experimenters were blinded to treatment groups during the study.

### The Vium Digital Platform

Vium Digital Smart Houses are outfitted with sensors that stream animal data and environmental conditions 24 h a day, 7 days a week. The digital platform collects, displays, and analyzes data on motion, breathing rate, temperature, humidity, illumination, and airflow continually in near real-time. This study used the activity metric. To compute activity, visible and infrared spectrum video is captured by dual cameras at 24 frames per second. Using computer vision, animal movement is read out as 0.024 frames/second as speed (mm/sec).

### Development of Digital Arthritis Index

The DAI was created and refined using data collected from a pilot experiment, then validated in vehicle-treated animals in two additional independent experiments (see Results section). Using computer vision analysis, video data from individual subjects was converted into continuous, high-temporal-resolution activity summaries. Video analysis revealed that, as rats became arthritic, not only movement, but also the maximum speed decreased. The DAI was therefore created to capture these changes. Salient features, including maximum speed of motion during the dark cycle, were extracted and aggregated by day for each individual animal. The aggregated data for a given day was then normalized to a baseline period (Study Days -2 to 5, removing induction day 0). The ratio of aggregated salient features to baseline salient features was termed the DAI. There is a negative relationship between DAI and overall activity: an increase in the DAI indicates an overall reduction in aggregated salient activity features on a specific study day.

### Detection and Measurement of Arthritis in Rats

Throughout the study, the DAI, body weights, ankle joint measurements, and clinical arthritis scores were assessed in individual animals to monitor disease progression. Ankle joint sizes (tibiotarsal joints) were determined using calipers (Swiss Precision Instruments, Garden Grove, CA, United States and Mitutoyo, Aurora, IL, United States) and were measured on induction day, day of booster, and daily during dosing. Based on previously published scoring methods (Brand et al., 2007; Hawkins et al., 2015), each hind paw was scored for the presence of inflammation and swelling, then summed to achieve the total arthritis score: 0 = normal, 1 = mild, but definite redness and swelling of ankle, or apparent redness and swelling limited to individual digits, regardless of number of affected digits, 2 = moderate redness and swelling of ankle, 3 = severe redness and swelling of the entire paw including digits, and 4 = maximally inflamed limb with involvement of multiple joints. Animals were euthanized 17 days post-induction by CO_2_ inhalation, followed by cervical dislocation. Ankle joints were harvested and stored in 10% neutral buffered saline until subsequent histopathology analysis.

Disease incidence, day of onset, and disease severity were calculated independently for all animals using ankle joint measurements, clinical arthritis scores, and the DAI. Based on internal experiments, animals were considered arthritic when the joint size, arthritis score, or DAI was greater than a threshold value. In the case of ankle joint size, the threshold was set at 0.25 inches. For arthritis scores, the threshold was set at 4, and in the case of DAI, the threshold was set at 5. Disease incidence was defined as the percentage of animals whose joint measurements, arthritis scores, or DAI were above the threshold during the 17-day study. Disease onset was defined as the earliest day in which ankle joint size, arthritis score, or DAI was consistently above the predetermined threshold. Disease severity was represented by the DAI or arthritis score value taken on the last study day (Day 17) or at endpoint if the animal was euthanized early for humane reasons. For joint measurements, disease severity was calculated using the change of joint size between the last study day/endpoint and baseline (induction day). Criteria for humane endpoint euthanasia included rear paw volumes greater than or equal to 0.4 inches, ulcerations or necrotic tissue at injection site for greater than 48 h, self-mutilation (causes were discussed with veterinarian), body weight reduction >20%, clinical score of 4 or any joint for greater than 48 h.

### Histopathologic Analysis of Tissues

Detailed reviews of the histological procedures have been previously described (Bolon et al., 2011; Caplazi et al., 2015). Briefly, after fixation, the tibiotarsal joints were transected at the level of the medial and lateral malleolus. Sagittal hemisections were cut to represent both larger (tarsal and carpal) and smaller (digital) joints for histological analysis, which was conducted by a board-certified pathologist blinded to treatment conditions (Bolder BioPath, Boulder, CO, United States). All joints were fixed in 10% neutral buffered formalin and placed in Surgipath decalcifier (Leica Biosystems; Buffalo Grove, IL, United States) for 1 week. Sections of joints were stained with hematoxylin and eosin (H&E) for a general histological evaluation and with toluidine blue to evaluate any cartilage pathology. Scores from 0 to 5 were given for each joint with respect to degree of inflammation, pannus formation, cartilage damage, bone resorption, and periosteal bone formation as previously described (Bendele et al., 1999). The total histopathology score is the sum of these individual scores for a total score of 25. Both the right and left hind joints were scored separately, then the mean score was calculated.

### Statistical Analysis

Two-way ANOVAs were used to confirm induction and investigate efficacy of drugs with treatment groups and study days as the independent variables. Follow-up pairwise comparisons were made using Tukey’s or Dunn’s tests for multiple comparisons for parametric and non-parametric statistical analyses, respectively. Unpaired *t*-tests or one-way ANOVAs were used to assess differences in average night-time motion, ankle joint severity scores, DAI severity scores, scores at day of onset, and cumulative arthritis scores, between two or more groups, respectively. Mann–Whitney and Kruskal–Wallis tests were used to assess day of onset, arthritis severity scores, and histopathology scores between two or more groups, respectively. Pearson tests were used to perform cross-correlation analyses. Linear regression analyses were performed with DAI, joint sizes, and arthritis scores as independent variables and total histopathological score as the dependent variable. *P*-values less than 0.05 were considered statistically different.

## Results

### The Vium Digital Platform Acquires Behavioral Measurements of Disease

We hypothesized that sensor-enabled Vium Digital Smart Housing can be used to create a method allowing for remote tracking of disease course. As a proof-of-concept, we used the collagen-induced arthritis (CIA) rat model of Rheumatoid Arthritis (RA). Disease was induced in rats (CIA Vehicle or “induced”) on Day 0 and a booster administered on Day 7, while control animals (Control Vehicle or “non-induced”) were injected with incomplete adjuvant alone (**Figure 1A** for schematic including the timing of vehicle treatment). We first confirmed effective disease induction using standard methods to assess disease progression, including joint size measurements, clinical arthritis scores, and body weight. As expected, ankle joint sizes, arthritis scores, and body weight were significantly altered in CIA compared to Control Vehicle rats over certain days post-induction [treatment group × study days, *F*_(10,171)_ = 22.53, *P* < 0.0001, *F*_(9,156)_ = 27.66, *P* < 0.0001, *F*_(10,171)_ = 3.95, *P* < 0.0001, respectively] (**Figures 1B–D**). Follow-up pairwise comparisons revealed that ankle joint size and arthritis scores were significantly increased in induced compared to non-induced rats on Days 13–17 post-induction (*P* < 0.001 for ankle joint size and *P* < 0.05 for arthritis scores for Days 13–17 vs. Day 0), while body weight was significantly decreased on Days 15–17 post-induction (*P* < 0.05 for Days 15–17 vs. Day 0). Increased ankle joints and arthritis scores, which indicate ankle joint edema and inflammation, respectively, confirm effective disease induction in CIA rats as previously described in the literature (Bendele, 2001; Brand et al., 2007; Vincelette et al., 2007).

**FIGURE 2:**
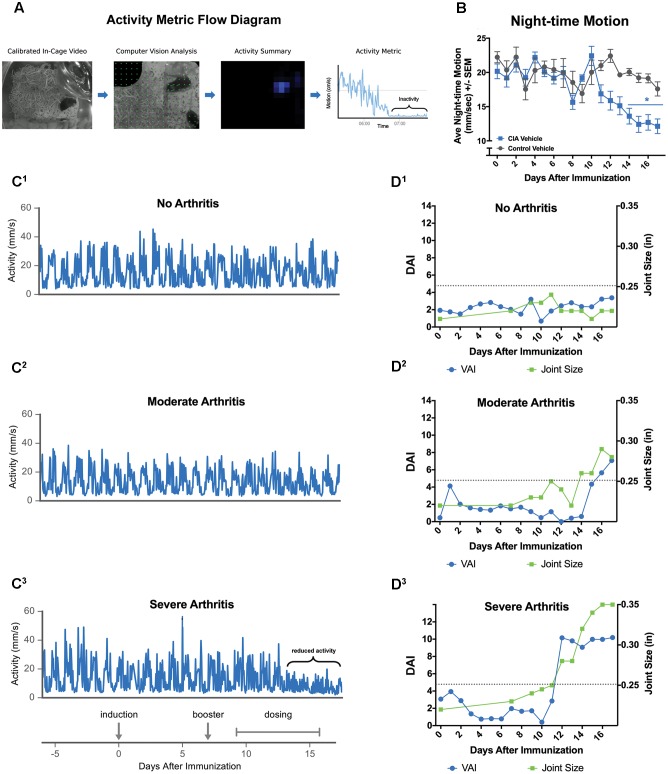
The Vium Digital Platform acquires behavioral measurements of disease. **(A)** Generation of automated activity metric: data processing flow demonstrating how streaming HD video of the animals is transformed into activity metrics using computer vision analysis. **(B)** CIA Vehicle (induced) rats showed significantly decreased night-time motion. **(C)** Representative plots showing activity (mm/s) for an animal with no arthritis (joint size < 0.25 inches or arthritis score of ≤2) **(C^1^)**, an animal with mild to moderate arthritis (joint size 0.25 to 0.30 inches or arthritis score of ≤6) **(C^2^)**, or severe arthritis (joint size > 0.30 inches or arthritis score of >6) **(C^3^)**. Decrease in overall activity was apparent only in the severely arthritic group (bracket in **C^3^**). Experiment timeline is aligned below. **(D^1^–D^3^)** The Digital Arthritis Index (DAI) plotted against the study day is shown for representative animals in **(C^1^–C^3^)**. The DAI (blue circles) tracked closely with maximum joint size (green squares), a conventional assessment method of arthritis. The arthritis threshold (dotted line) indicates that the DAI and ankle joint sizes are significantly different from controls, suggesting presence of arthritis. ^∗^*P* ≤ 0.05 vs. Control Vehicle. Error bars are ±SEM. *n* = 9/group.

Reduced mobility due to arthritis has been used to assess disease in rodent models of arthritis (Philippe et al., 1997; Vincelette et al., 2007; Mossiat et al., 2015; Tanimoto et al., 2015; Hayer et al., 2016); therefore, we took advantage of the ability of the Vium Digital Platform to automatically collect activity metrics. Motion (mm/s) was calculated using computer vision algorithms and was recorded continuously for each rat throughout the experiment (**Figure 2A** and Methods). From raw motion, average daily daytime and night-time motion were calculated. We focused on night-time motion as rodents are more active during the dark cycle (**Figure 2B**). Prior to induction, dark cycle activity was indistinguishable between the groups. Over study days -3 to -1, CIA Vehicle and Control Vehicle animals had average nightly motion of 22.3 ± 2.4 and 21.7 ± 2.3 mm/s, respectively [*F*_(8,7)_ = 1.15, ns]. We assessed whether changes in gross dark cycle activity would be associated with disease progression. Both CIA Vehicle and Control Vehicle animals showed a significant decrease in motion between days 13 and 17 compared to days -3 to -1 (baseline) [*F*_(1,30)_ = 62.93, *P* < 0.0001]. Non-induced animals exhibited a mild, yet statistically significant 14% decrease in average dark cycle activity between days 13 and 17 and baseline (18.6 ± 1.5 mm/s compared to 21.7 ± 2.3 mm/s, *P* < 0.05). In comparison, induced animals exhibited a more pronounced 43% decrease in activity over the Day 13 to 17 interval compared to baseline (12.4 ± 3.0 mm/s compared to 22.3 ± 2.4 mm/s, *P* < 0.0001) (**Figures 2B,C**). Comparing activity levels for individual animals revealed that 8 of 9 induced rats showed greater than 15% reductions in activity compared to 3 of 8 non-induced rats. The single CIA Vehicle or induced rat that did not show a decrease in activity was found to have moderate arthritis (joint size = 0.28 inches, arthritis score = 5, histopathology sum score = 3.75) (**Figures 2C^2^,D^2^**). These data suggest that gross motion, as detected by the Vium Digital Platform, can be used to indirectly assess animal health and wellness. However, gross motion metrics did not appear sensitive enough to detect finer aspects of RA disease, including incidence and severity. To address this issue, we developed an automated index, which we termed the DAI.

### The Digital Arthritis Index (DAI) Detects Development of Arthritis

The DAI measures specific features of motion in order to capture disease progression in RA, such that increases in the DAI indicate increasing disease severity (see Materials and Methods for details on development of DAI). In **Figure 2D**, the DAI is plotted together with joint size measurements for the same representative rats shown in **Figure 2C**. Similar to joint measurements, the DAI was increased for rats with both moderate and severe arthritis compared to controls, indicating the presence of disease. These data suggest that the DAI may be more sensitive than gross night-time motion and led us to further investigate its potential as a low-touch metric to detect disease and differentiate varying levels of severity.

**FIGURE 3:**
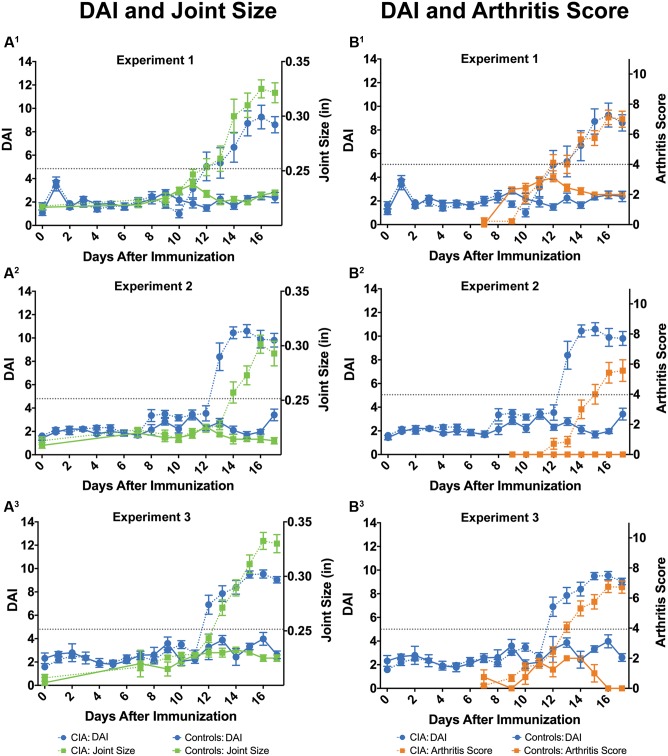
The DAI reproducibly detects disease similar to standard methods. In three independent experiments, the DAI (blue circles) tracked with maximum joint size (green squares) **(A^1^–A^3^)** and arthritis scores (orange squares) **(B^1^–B^3^)**, two standard *in vivo* methods to assess disease. Similar to standard methods, the DAI detected arthritis in CIA-induced rats (dotted lines) compared to non-induced controls (solid lines). Arthritis threshold (dotted gray line) indicates that DAI, ankle joint sizes, and arthritis scores are significantly different from controls, suggesting presence of arthritis (*P* ≤ 0.05). Error bars are ±SEM. For Experiment 1, *n* = 9/group. For Experiment 2, *n* = 7/group. For Experiment 3, *n* = 4 for CIA rats and *n* = 16 for Control rats.

To demonstrate that the DAI can detect development of arthritis, we compared the DAI to ankle joint measurements and clinical arthritis scores across three independent CIA experiments (**Figure 3** and **Table 1**). In the first experiment, ankle joint measurements were significantly increased in CIA Vehicle rats over days 13 to 17 post-induction [treatment group × study days, *F*_(10,160)_ = 41.71, *P* < 0.0001] (**Figure 3A^1^**). Similarly, arthritis scores were significantly different from Control Vehicle rats on days 13–17 post-induction [treatment group × study days, *F*_(9,156)_ = 27.66, *P* < 0.0001] (**Figure 3B^1^**). In comparison, the DAI was significantly increased in CIA Vehicle rats compared to control rats from days 12 to 17 post-induction [treatment group × study days, *F*_(17,266)_ = 11.32, *P* < 0.0001] (**Figures 3A^1^,B^1^**). Similar results were observed in the second and third experiments: the DAI detected significant differences between vehicle-treated CIA and Control rats, and these changes paralleled ankle joint measurements and arthritis scores. In the second experiment, the DAI significantly increased from days 13 to 17 post-immunization [treatment group × study days, *F*_(17,216)_ = 35.01, *P* < 0.0001], whereas ankle joint measurements and arthritis scores significantly increased from days 14 to 17 [treatment group × study days, *F*_(10,120)_ = 28.32, *P* < 0.0001 and *F*_(8,108)_ = 24.36, *P* < 0.0001, respectively] (**Figures 3A^2^,B^2^**). In the third experiment, the DAI significantly increased from days 12 to 17 [treatment group × study days, *F*_(17,319)_ = 11.71, *P* < 0.0001], while ankle joint measurements and arthritis scores significantly increased from days 13 to 17 post-immunization [treatment group × study days, *F*_(10,180)_ = 22.78, *P* < 0.0001 and *F*_(9,175)_ = 14.75, *P* < 0.0001] (**Figures 3A^3^,B^3^**). Histopathological assessment of ankle joints from subjects in all three experiments revealed significantly increased histopathology scores for CIA Vehicle compared to Control Vehicle rats, indicating joint damage (**Table 1** and Supplementary Table 1).

**Table 1:** Validation of the Digital Arthritis Index (DAI) in three independent experiments.

	Experiment 1		Experiment 2		Experiment 3	
			
	CIA	Control	CIA	Control	CIA	Control
**Digital Arthritis Index (DAI)**						
Incidence	100% (9 of 9)	0% (0 of 9)	100% (7 of 7)	0% (0 of 7)	100% (16 of 16)	0% (0 of 4)
Onset	13.3 ± 1.7	N/A	13.0 ± 0.6	N/A	12.6 ± 0.9	N/A
Severity	9.7 ± 1.8^∗^	3.1 ± 1.2	10.5 ± 1.6^∗^	4.1 ± 1.3	9.5 ± 1.4^∗^	0.8 ± 0.6
**Joint size**						
Incidence	100% (9 of 9)	0% (0 of 9)	100% (7 of 7)	0% (0 of 7)	100% (16 of 16)	0% (0 of 4)
Onset	12.8 ± 1.3	N/A	14.6 ± 0.8^∧^	N/A	13.0 ± 1.2	N/A
Δ Size (in)	0.11 ± 0.03^∗^	0.01 ± 0.009	0.07 ± 0.03^∗^	-0.007 ± 0.02	0.12 ± 0.02^∗^	0.005 ± 0.03
**Arthritis score**						
Incidence	100% (9 of 9)	0% (0 of 9)	71% (5 of 7)	0% (0 of 7)	94% (15 of 16)	0% (0 of 4)
Onset	13.7 ± 1.6	N/A	14.8 ± 0.8^∧^	N/A	14.2 ± 1.4^∧^	N/A
Severity	7.2 ± 1.3^∗^	2.0 ± 0	5.6 ± 1.9^∗^	0	7.1 ± 1.3^∗^	0
**Histopathology**						
Score	7.4 ± 2.8^∗^	0.14 ± 0.25	8.8 ± 2.6^∗^	0	10.8 ± 2.4^∗^	0


*Disease incidence, onset, and severity for CIA and control rats were calculated using the DAI, ankle joint size measurements, and arthritis scores. Values represent means ± std. ^∗^*P* ≤ 0.001 vs. control, ^∧^*P* ≤ 0.05 vs. DAI onset.*

Ankle joint measurements and arthritis scores represent standard *in vivo* methods by which disease trajectory, day of onset, and severity (including incidence) of CIA are determined (Vincelette et al., 2007). We therefore assessed the ability of the DAI to determine disease trajectory similar to conventional methods (**Table 1**). The incidence of arthritic rats was similar between DAI and standard methods. The DAI and ankle joint measurements identified 100% of the CIA rats (32 of 32) and no control rats (0 of 20) across the three experiments. In comparison, arthritis scores identified 91% of the CIA rats (29 of 32) and no control rats (0 of 20). The average day to disease onset was similar between the DAI and standard methods; however, there were small, yet significant differences in Experiments 2 and 3, wherein the DAI detected disease onset approximately a day earlier compared to standard methods (See above results and **Table 1**). At study endpoint, the DAI, joint measurements, and arthritis scores were significantly increased in CIA Vehicle compared to Control Vehicle animals, and these observations were reflected in the histopathology scores (**Table 1**, Supplementary Table 1 and Figure 1C).

To assess reproducibility of the DAI, we compared disease trajectory, as measured by the DAI, across all three independent studies (**Table 1**). The day of disease onset was not significantly different across studies (*H* = 2.65, ns), as well as the DAI values at disease onset [Experiment 1: 7.0 ± 1.6, Experiment 2: 8.9 ± 1.8, Experiment 3: 8.2 ± 2.1, *F*_(2,29)_ = 2.04, ns]. Furthermore, the DAI at study endpoint (severity) was also similar across studies [*F*_(2,27)_ = 0.62, ns].

Collapsing studies, we compared disease severity by cross-correlation analyses between the DAI and standard *in vivo* methods (joint size and arthritis scores), as well as between the DAI and ankle joint histopathology, the gold standard for disease assessment (Seeuws et al., 2010; Scales et al., 2016) (**Table 2**). Compared to standard *in vivo* methods, the DAI positively correlated with joint size and arthritis scores (*R* = 0.84 and 0.87, respectively, *P* < 0.0001). The DAI also showed a positive correlation with a number of histopathological assessments, including degree of joint inflammation (*R* = 0.93, *P* < 0.0001), a component of the total histopathology score. Furthermore, the DAI displayed a slightly stronger positive correlation to the total histopathology score than did joint size or arthritis score (*R* = 0.91 for DAI, *R* = 0.87 for joint sizes, and *R* = 0.89 for arthritis scores, *P* < 0.0001 for all). Additional linear regression analyses corroborated these results; total histopathological score is more strongly predicted by the DAI (*R*^2^ = 0.82 for DAI, *R*^2^ = 0.76 for joint sizes, and *R*^2^ = 0.80 for arthritis scores sizes, *P* < 0.0001 for all). In summary, these results reveal that similar to standard *in vivo* methods, the DAI reproducibly detects changes in disease progression in a standard CIA rat model of RA. Moreover, the DAI correlates with underlying disease pathology and standard methods for assessing RA disease progression.

**Table 2:** Cross-correlation analysis between Digital Arthritis Index (DAI) and standard measures.

	DAI	Δ Joint size	Arthritis score
DAI		0.84*	0.87*
Δ Joint size	0.84^∗^		0.90*
Arthritis Score	0.87^∗^	0.90*	
Histopathology			
Inflammation score	0.93^∗^	0.89*	0.94*
Pannus	0.79^∗^	0.83*	0.78*
Cartilage damage	0.89^∗^	0.81**	0.87*
Bone resorption	0.78^∗^	0.82*	0.77*
Periosteal bone formation	0.71^∗^	0.64*	0.62*
Total score	0.91^∗^	0.87*	0.89*


**n* = 52 rats from three independent experiments were used for analysis. ^∗^*P ≤* 0.0001.*

### Digital Arthritis Index Detects Response to Standard-of-Care RA Drugs

**FIGURE 4:**
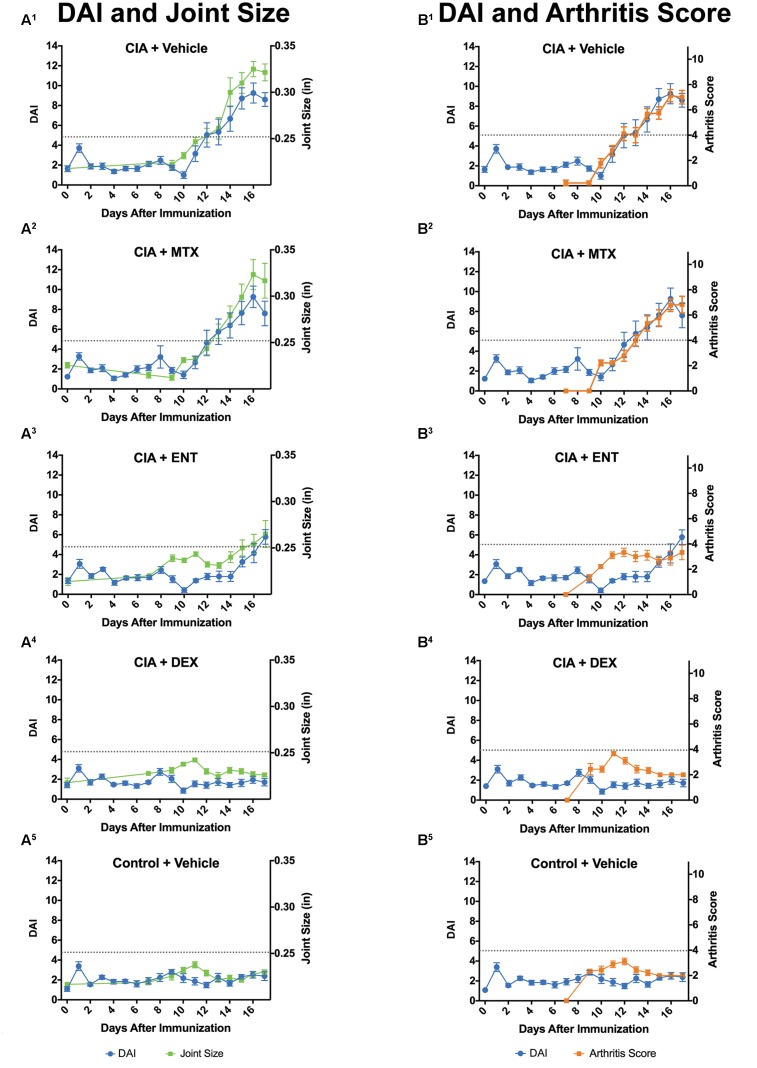
The DAI detects response to standard of care (SOC) drugs. CIA-induced rats were treated with vehicle, methotrexate (MTX), etanercept (ENT), or dexamethasone (DEX). For all SOC drugs, the DAI (blue circles) tracked closely with joint size (green squares) **(A^1^–A^5^)** and arthritis scores (orange squares) **(B^1^–B^5^)**. Arthritis threshold (dotted gray line) indicates that DAI, ankle joint sizes, and arthritis scores are significantly different from controls, suggesting presence of arthritis (*P* ≤ 0.05). Error bars are ±SEM. *n* = 9/group.

We next investigated whether the DAI can detect efficacy of three SOC RA drugs: methotrexate (MTX), etanercept (ETN), and dexamethasone (DEX) (Hegen et al., 2008). As in the previous validation studies, we compared the DAI to standard methods, including ankle joint measurements, arthritis scores, body weights, and ankle joint histopathology (**Figure 4**, **Table 3** and Supplementary Figure 1).

**Table 3:** Summary statistics for disease course with standard of care (SOC) drugs.

	Vehicle	MTX	ENT	DEX
**Digital Arthritis Index (DAI)**				
Incidence	100% (9 of 9)	89% (8 of 9)	78% (7 of 9)^∧^	0% (0 of 9)
Onset	13.3 ± 1.7	13.0 ± 1.8	16.1 ± 0.9^∗^	N/A
Severity	9.7 ± 1.8	9.7 ± 3.3	6.8 ± 2.1^∗^	2.4 ± 1.1^∗^
**Joint size**				
Incidence	100% (9 of 9)	89% (8 of 9)	56% (5 of 9)	0% (0 of 9)
Onset	13.0 ± 1.2	13.3 ± 1.4	15.8 ± 1.3^∗^	N/A
Δ size (in)	0.11 ± 0.03	0.12 ± 0.05	0.05 ± 0.04^∗^	-0.001 ± 0.006^∗^
**Arthritis score**				
Incidence	100% (9 of 9)	89% (8 of 9)	22% (2 of 9)	0% (0 of 9)
Onset	13.7 ± 1.6	14.0 ± 1.5	15.0 ± 2.8	N/A
Severity	7.2 ± 1.3	7.1 ± 1.4	3.3 ± 1.7^∗^	2.0 ± 0^∗^
**Histopathology**				
Score	7.4 ± 2.8	8.1 ± 3.8	2.6 ± 2.7^∗^	0.1 ± 0.3^∗^


*Disease incidence, onset, and severity were calculated using the DAI, ankle joint size measurements, and arthritis scores for CIA-induced rats treated with SOC drugs. Values represent means ± std. ^∗^*P* ≤ 0.01 vs. CIA Vehicle-treated group.*

*^∧^The DAI identified two additional ENT-treated rats with arthritis that were not identified via conventional joint measurements. Histopathology scores for the rats were 0.5 and 1.5, indicating very mild disease.*

Ankle joint measurements and arthritis scores were significantly altered in DEX-treated rats. [treatment group × study days, *F*_(10,171)_ = 23.38, *P* < 0.0001 and *F*_(9,156)_ = 27.77, respectively, *P* < 0.0001], ENT-treated rats [treatment group × study days, *F*_(10,170)_ = 8.64 and *F*_(9,156)_ = 10.68, respectively, *P* < 0.0001], but not MTX-treated rats [treatment group × study days, *F*_(10,168)_ = 0.43 and *F*_(9,153)_ = 0.60, respectively, ns] (**Figures 4A,B**). Values were decreased on Days 13–17 for DEX-treated rats and Days 14–17 for ENT-treated rats compared to CIA Vehicle rats (*Ps* < 0.05 and *P*s < 0.001, respectively). In comparison, the DAI was also significantly altered in DEX-treated rats [*F*_(17,280)_ = 13.41, *P* < 0.0001], ENT-treated rats [F_(17,284)_ = 5.60, *P* < 0.0001], but not MTX-treated rats [*F*_(17,281)_ = 0.20, ns] (**Figures 4A,B**). The DAI decreased on Days 12–17 for DEX-treated rats and Days 12–16 for ENT-treated rats compared to CIA Vehicle rats (*P*s < 0.0001 and *P*s < 0.01, respectively). The cumulative arthritis index over threshold confirmed significant differences for DEX- and ENT-treated rats compared to CIA vehicle rats (*P* < 0.0001 and *P* < 0.05, respectively) (Supplementary Figure 1A). Unlike joint measurements, arthritis scores, and the DAI, body weight measurements showed varying changes across SOC treatment groups (Supplementary Figure 1B). When compared to CIA Vehicle rats, only ENT-treated rats showed significantly improved body weights on Days 13–17 (*P*s < 0.05). In contrast, MTX had no effect, and DEX-treated rats demonstrated a significant decrease in body weight on Days 11 and 14 (*P*s < 0.05).

We also assessed the ability of the DAI to discern disease trajectory among SOC compounds (**Table 3**). The DAI determined the incidence of arthritis in a similar percentage of animals for vehicle, DEX, ENT, and MTX-treated animals as standard methods. The DAI, ankle joint measurements, and arthritis scores identified 89% (8 of 9) of the MTX-treated rats and none (0 of 9) of the DEX-treated rats as having disease. The DAI identified 78% (7 of 9) ENT-treated rats to be arthritic compared to joint size measurements and arthritis scores, which identified 56% (5 of 9) and 22% (2 of 9) of treated rats, respectively.

In addition to identifying diseased animals, the DAI detected SOC compounds that delayed disease onset. The DAI detected delayed onset in ENT-treated compared to CIA Vehicle rats (*H* = 10.83, *P* < 0.01) (**Table 3**). Delayed onset was confirmed by joint size measurements (*H* = 8.81, *P* < 0.01), but not by arthritis scoring (*H* = 0.47, ns); however, the latter metric was not statistically significant due to the low incidence of diseased rats (*n* = 2). For MTX, there was no difference between the average days to disease onset between DAI and standard measurements (*H* = 2.00, ns).

At study endpoint, there was a significant main effect of disease severity across SOC compounds as measured by the DAI [*F*_(3,32)_ = 21.67, *P* < 0.0001], joint size measurements [*F*_(3,32)_ = 7.15, *P* < 0.001] and arthritis scoring (*H* = 27.28, *P* < 0.0001) (**Table 3**). Follow-up pairwise comparisons demonstrated that disease severity in DEX-treated rats was significantly lower than other groups as measured by either DAI (*P* < 0.001 vs. Vehicle, MTX, and ENT), joint size (*P* < 0.05 vs. Vehicle, MTX, and ENT), or arthritis scores (*P* < 0.01 vs. Vehicle and MTX). The ENT-treated rats also demonstrated improvements in disease severity when indexed by the DAI or arthritis scores (DAI: *P* < 0.05 vs. Vehicle and MTX, arthritis scores: *P* < 0.05 vs. Vehicle and MTX). However, joint size measurements did not reach statistical significance. Overall, the DAI, joint measurements, and arthritis scores ranked the effects of the SOC compounds as follows: DEX > ENT > MTX > Vehicle. Joint histopathology scores, generally regarded as the gold standard to determine efficacy, placed agents in a similar rank order; DEX > ENT > MTX > Vehicle (**Table 3** and Supplementary Figure 1C). In summary, the DAI detected the response of SOCs similar to both standard *in vivo* and histopathology methods.

### The Digital Arthritis Index Determines Response for Nine Compounds

**FIGURE 5:**
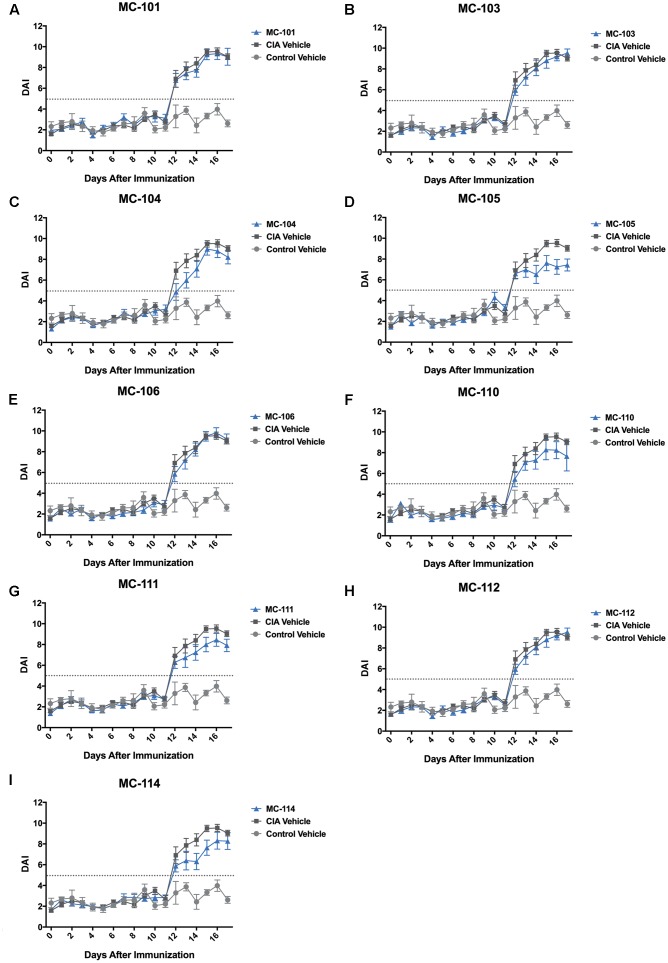
The DAI detects therapeutic response in repurposed compounds. **(A–I)** The DAI detected varying responses and improvements in Compounds MC-103, MC-104, MC-105, MC-110, MC-111, and MC-114 compared to CIA Vehicle rats {Significant main effect of dose for MC-103 [*F*_(1,462)_ = 7.17, *P* < 0.01], MC-104 [*F*_(1,462)_ = 10.29, *P* < 0.01], MC-105 [*F*_(1,463)_ = 11.51, *P* = 0.001], MC-110 [*F*_(1,459)_ = 11.38, *P* < 0.001], MC-111 [*F*_(1,463)_ = 9.96, *P* < 0.01], and MC-114 [*F*_(1,463)_ = 10.66, *P* < 0.01]}. In this analysis, all doses were combined. Arthritis threshold (dotted gray line) indicates that DAI is significantly different from controls, suggesting presence of arthritis (*P* ≤ 0.05). Error bars are ±SEM. For each compound, *n* = 12. For CIA Vehicle rats, *n* = 4. For Control Vehicle rats, *n* = 16.

**FIGURE 6:**
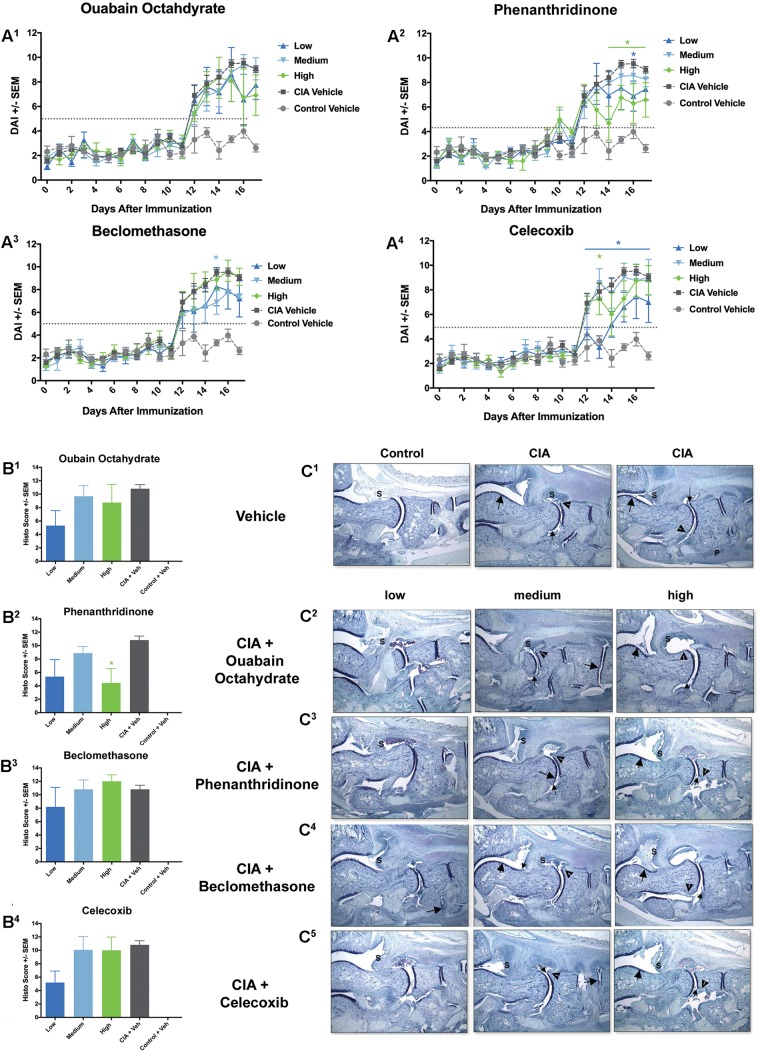
A subset of compounds demonstrates dose-dependent responses. **(A)** DAI: Compared to CIA Vehicle rats, rats treated with the high and lose dose of ouabain octahydrate showed trends toward decreased DAI at study end **(A^1^)**. There was also significant reduction in the DAI for rats treated with the high and low doses of phenanthridinone **(A^2^)**, the low dose of beclomethasone **(A^3^)**, and in the low dose of celecoxib **(A^4^)** (^∗^*P* ≤ 0.05). Arthritis threshold (dotted gray line) indicates the threshold at which the DAI is significantly different from controls, suggesting presence of arthritis. CIA Vehicle rats showed significantly elevated DAI scores compared to Control Vehicle rats (*P* ≤ 0.05). **(B)** Histopathology scores: Ouabain octahydrate **(B^1^)**, phenanthridinone **(B^2^)**, beclomethasone **(B^3^)**, and celecoxib **(B^4^)** showed dose-dependent trends; however, only the high dose of phenanthridinone was significantly reduced compared to CIA Vehicle rats (CIA + Veh) (^∗^*P* ≤ 0.05). CIA + Veh rats showed significantly elevated histopathology scores compared to Controls (Control + Veh) (*P* ≤ 0.05). **(C)** Representative ankle joint histopathology images. **(C^1^)** Left panel: Vehicle-treated control rat showed a normal synovium (S). Center and Right panels: Vehicle-treated, CIA-induced rats showed severe inflammation in synovium (S), mild to moderate cartilage damage (large arrow), mild to minimal pannus (small arrow), mild to minimal bone resorption (arrowhead), and some evidence of periosteal bone formation (P). **(C^2^)** Rat treated with the low dose of ouabain octahydrate (left panel) showed mild inflammation in synovium (S) with very minimal cartilage damage and pannus. **(C^3^)** Rats treated with low and high doses of phenanthridinone (left and right panels) showed reduced inflammation in synovium, minimal cartilage damage, pannus, and bone resorption. **(C^4^)** Rat treated with the lowest dose of beclomethasone (left panel) showed severe inflammation, mild cartilage damage with very minimal pannus and bone resorption. **(C^5^)** Rat treated with lowest dose of celecoxib (left panel) showed reduced inflammation and very minimal cartilage damage, pannus, and bone resorption. S = synovium (indicating area of possible inflammation), large arrow = cartilage damage, small arrow = pannus, arrowhead = bone resorption, P = periosteal bone formation. Note that pannus, bone resorption, and periosteal bone formation are not always evident in 16× magnification). Error bars are ±SEM. For each dosing group, *n* = 4. For CIA Vehicle rats, *n* = 4. For Control Vehicle rats, *n* = 16.

Preclinical drug discovery relies not only on the reproducibility of assessment methods, but also on the ability of standardized methods to assess therapeutic efficacy (Prior et al., 2014). We next explored the accuracy of the DAI for determining the potential efficacy of nine compounds repurposed by the SMarTR^TM^ Engine for RA. We first assessed overall efficacy for each drug across three doses and found six compounds (of the nine compounds assessed) that demonstrated overall significant main effects of treatment when compared to CIA Vehicle (**Figure 5** and Supplementary Figure 2). We then selected four significant compounds for further analysis, namely, ouabain octahydrate, phenanthridinone, beclomethasone, and celecoxib (**Figure 6** and Supplementary Figure 3).

We assessed compounds based on three different *in vivo* methods: the traditional joint size measurements or arthritis scores or the novel DAI, which were then compared to histopathology (**Table 4**). We used total histopathology scores and inflammation scores, the component of the histopathology score that showed the highest correlation among all three *in vivo* methods (**Table 3**). We ranked compounds from highest to lowest according to which agents produced the largest to lowest reductions, indicating disease attenuation, in the specified methods. We found that all *in vivo* and histopathology assessment methods were in agreement, ranking MC-105 as the compound that produced the largest reduction, thereby demonstrating the most potential for therapeutic efficacy. Interestingly, the rank order produced from the total histopathology scores most closely resembled the rank order produced from the DAI, while the rank order resulting from the inflammation scores most closely resembled those produced from joint size measurements and arthritis scores. Compound MC-114 exemplified this differential sensitivity. MC-114 was ranked either first or second when employing inflammation scores, joint size measurements, or arthritis scores. However, when using total histopathology score or the DAI, MC-114 was not ranked as highly (fourth). Only one compound (MC-111) showed dissociation between histopathology scores and *in vivo* methods. MC-111 was ranked one of the lowest compounds by histopathology; yet *in vivo* measurements ranked this compound higher than did histopathology. Overall, all three *in vivo* methods performed similarly in detecting response of compounds.

**Table 4:** Summary of therapeutic response of nine repurposed compounds by method of arthritis assessment.

Compound	Total score (histopathology)	Digital Arthritis Index	Inflammation score (histopathology)	Arthritis score	Δ Joint size (in)
MC-105	6.3 ± 4.1** (1)	7.4 ± 2.0** (1)	3.02 ± 0.46** (1)	4.0 ± 2.6 *** (1)	0.08 ± 0.05* (1)
MC-103	7.9 ± 4.5 (2)	7.9 ± 2.5 (2)	3.67 ± 0.36 (3)	5.3 ± 3.3 (3)	0.11 ± 0.06 (3)
MC-110	8.2 ± 4.1 (3)	8.3 ± 3.4 (4)	3.81 ± 0.28 (4)	5.8 ± 2.6 (4)	0.11 ± 0.04 (3)
MC-114	8.4 ± 4.2 (4)	8.3 ± 2.8 (4)	3.60 ± 0.52* (2)	4.3 ± 2.1*** (2)	0.08 ± 0.04*** (1)
MC-104	8.9 ± 3.6 (5)	8.4 ± 2.1 (6)	4.06 ± 0.37 (5)	5.8 ± 2.5 (4)	0.11 ± 0.04 (3)
MC-106	9.3 ± 2.6 (6)	9.4 ± 1.6 (8)	4.25 ± 0.07 (6)	5.8 ± 1.7 (4)*	0.11 ± 0.03 (3)
MC-101	9.8 ± 3.4 (7)	9.0 ± 2.4 (7)	4.27 ± 0.42 (7)	6.3 ± 2.2 (8)	0.12 ± 0.05 (8)
MC-112	10.0 ± 2.9 (8)	9.5 ± 1.4 (9)	4.50 ± 0.22 (9)	6.4 ± 1.5 (9)	0.12 ± 0.04 (8)
MC-111	10.3 ± 3.9 (9)	7.9 ± 2.1* (2)	4.38 ± 0.51 (8)	6.1 ± 2.4 (7)	0.11 ± 0.04 (3)
Vehicle	10.8 ± 2.4	9.5 ± 1.4	4.75 ± 0.07	7.1 ± 1.3	0.13 ± 0.03


*Nine repurposed compounds at three doses each are ranked from highest (1) to lowest (9) according to which agent produced the largest to lowest reductions, respectively, based on the assessment method. For the purposes of this table, doses were combined. For each compound, *n* = 12 and for Vehicle, *n* = 16. ^∗^*P* ≤ 0.05 vs. vehicle, ^∗∗^*P* ≤ 0.01 vs. vehicle, ^∗∗∗^*P* ≤ 0.001 vs. vehicle.*

From the six compounds initially identified to have significantly different DAI scores compared to controls, we selected ouabain octahydrate (MC-103), phenanthridinone (MC-105), beclomethasone (MC-111), and celecoxib (MC-114) for further analysis (**Figure 6** and Supplementary Figure 3). Ouabain octahydrate and phenanthridinone showed attenuation of disease severity as determined by the DAI, standard measurements, and histopathology. Beclomethasone showed stronger attenuation by the DAI compared with arthritis scoring and histopathology. Celecoxib showed stronger attenuation by standard measurements compared with the DAI or histopathology.

For ouabain octahydrate, there was a trend for the DAI to show differences among dose groups [*F*_(3,426)_ = 2.42, *P* = 0.07], with rats treated with the low and high doses of the compound showing lower DAI values specifically at study end (**Figure 6A^1^**). Joint size measurements and arthritis scores demonstrated significant main effects for dose, with rats treated with the low and high dose of the compound trending toward lower joint size measurements and arthritis scores [*F*_(3,259)_ = 2.71, *P* < 0.05 and *F*_(3,235)_ = 2.73, *P* < 0.05, respectively] (Supplementary Figures 3A^1^,B^1^). There was a trend for low dose-treated rats to show reduced histopathology scores (**Figures 6B^1^,C^2^**).

For phenanthridinone, the DAI determined a significant main effect of dose [*F*_(3,427)_ = 4.69, *P* < 0.01], with the following dose effects: High > Low > Medium (**Figure 6A^2^**). Rats treated with the high and low doses showed lower DAI values compared to CIA Vehicle rats, but only high-dose treated rats reached statistical significance (*P* < 0.05). Joint size measurements and arthritis scores also demonstrated significant main effects of dose [*F*_(3,259)_ = 21.11, *P* < 0.0001 and *F*_(3,235)_ = 25.37, *P* < 0.0001, respectively] (Supplementary Figures 3A^2^,B^2^). High and low dose-treated rats showed significantly lower joint size measurements and arthritis scores compared to CIA Vehicle rats (*P* < 0.01 and *P* < 0.001, respectively). Histopathology corroborated these results; both high and low dose-treated rats showed reduced histopathology scores; however, only rats treated with the high dose of phenanthridinone reached statistical significance (*P* < 0.05 vs. CIA Vehicle) (**Figures 6B^2^,C^3^**).

For beclomethasone, the DAI determined a significant main effect of dose [*F*_(3,427)_ = 6.14, *P* < 0.001], with the following dose effects: Medium > Low > High (**Figure 6A**^3^). Rats treated with the medium and low doses showed significantly lower DAI values compared to CIA Vehicle rats (*P*s < 0.05 for all). Joint size measurements and arthritis scores also demonstrated significant main effects of dose, though with the following dose effects: Low > Medium > High [*F*_(3,259)_ = 4.84, *P* < 0.01 and *F*_(3,235)_ = 5.09, *P* < 0.01, respectively] (Supplementary Figures 3A^3^,B^3^). Compared to CIA Vehicle rats, low-dose treated animals had significantly lower joint size measurements and arthritis scores (*P*s < 0.01 for all). Similar to standard measurements, there was a trend for rats treated with the lowest dose of beclomethasone to show slightly reduced histopathology scores, while rats treated with the medium and high doses were mostly similar to disease controls (**Figures 6B^3^,C^4^**).

Finally, for celecoxib, the DAI determined a significant main effect of dose [*F*_(3,427)_ = 6.77, *P* < 0.001], with the following dose effects: Low > High > Medium (**Figure 6A^4^**). Rats treated with the lowest dose showed significantly reduced DAI compared to CIA Vehicle rats (*P* < 0.001). Joint size measurements and arthritis scores also demonstrated significant main effects of dose [*F*_(3,259)_ = 18.17, *P* < 0.0001 and *F*_(3,235)_ = 20.48, *P* < 0.0001, respectively] (Supplementary Figures 3A^4^,B^4^). Compared to CIA Vehicle rats, low and high dose-treated rats had significantly lower joint size measurements and arthritis scores (*P*s < 0.01 for all). Similar to the DAI, there was a trend for rats treated with the lowest dose of celecoxib to show reduced histopathology scores while the medium and high doses were mostly similar to disease controls (**Figures 6B^4^,C^5^**).

## Discussion

Herein, we demonstrated the utility of a low-touch and reproducible digital platform for phenotyping animal models of disease. As a proof-of-concept, we used the CIA rodent model of RA, a well-established preclinical model broadly applied in rheumatology research (Bendele, 2001; Bevaart et al., 2010; Bolon et al., 2011), to test the reproducibility and predictive validity of this digital platform in automatically tracking disease. The resultant DAI determines disease progression similar, and at times earlier, compared with standard methods across multiple studies. Furthermore, the DAI: (1) detects different patterns of therapeutic response to SOC RA drugs, (2) strongly correlates with disease histopathology, and (3) complements standard methods to determine response in a phenotypic screen of repurposed compounds. Our results confirm effective disease induction and are consistent with previously published data (Bendele, 2001; Hegen et al., 2008). Although the SOC drug methotrexate (MTX) did not ameliorate symptoms of arthritis in the current study, this is likely due to the lower dose used for this study compared to previous studies (0.075 mg/kg vs. >0.1 mg/kg) (Sakuma et al., 2001).

Standard *in vivo* techniques to assess arthritis, including ankle joint size measurements and clinical arthritis scoring, are indicative of inflammation, erythema, and edema (Bendele, 2001; Brand et al., 2007). Although these techniques are widely accepted and used in the field, there are some limitations: the semi-quantitative nature of the clinical arthritis scores and the inability of these techniques to reflect more functional aspects of disease, including mobility, particularly in later phases of disease (Welsing et al., 2001; Song et al., 2015). RA is characterized by immune-mediated inflammatory responses in the synovial membrane, accompanied by the infiltration of a variety of mononuclear cells in the early stages of disease progression (Firestein, 2003). Chronic inflammation and abnormal proliferation ultimately form a destructive pannus, which progressively destroys the cartilage and bone in joints. Infiltrating immune cells secrete various cytokines and chemokines, such as the Receptor Activator of Nuclear factor Kappa-B (RANK) ligand (RANKL). These contribute to the inflammation of the synovial membrane and stimulate osteoclastogenesis and bone remodeling (Firestein, 2003). As disease progresses, signs and symptoms of inflammation may decrease despite continued joint destruction (Song et al., 2015). The degree of inflammation early in disease and severity of joint damage later in disease are predictive of decreased functional capacity in RA patients (Welsing et al., 2001). For patients with chronic disease, cartilage damage and bone erosion have been shown to be more prominent determinants of functional impairment than are indices of synovial inflammation (Hayer et al., 2016). Therefore, preventing bone damage is an important target for developing novel anti-arthritic agents. Conversely, treatments that reverse synovial inflammatory processes do not necessarily lead to full recovery of joint functionality (Bombardier et al., 2012; Hayer et al., 2016). These observations underscore the importance of platforms that not only objectively quantify the inflammatory aspects of the disease, but that are also sensitive to determining the degree of joint damage, as the latter will reflect long-term functional capacity during disease.

A growing body of literature highlights the significance of performing objective, activity-based analysis in animal models of RA. Spontaneous locomotion, gait analysis, and biotelemetry have also provided novel insights for current therapeutic compounds (Philippe et al., 1997; Sasakawa et al., 2005; Vincelette et al., 2007; Mossiat et al., 2015; Tanimoto et al., 2015; Hayer et al., 2016). However, current activity-based behavioral assays do have limitations. These assays require assessment of animals outside of the home cage or implantation with telemetry devices. Additionally, they are time-consuming procedures, which may be restrictive to users and limit the frequency of sampling behavioral time points. In comparison, the DAI reflects the objective and functional nature of these activity-based behavioral assays while providing the consistency and convenience of an automated metric that can be collected daily from animals housed in their home cages, subsequently reducing animal handling and other potential “stressors” that may affect study reproducibility. These features are useful for longer duration experiments that investigate beyond acute inflammation and that examine the effects of cartilage damage and joint destruction.

In this study, we reveal that the DAI not only detects disease progression similar to standard measurements, but also correlates well with histopathology methods. The DAI more closely paralleled total histopathology scores than do manual joint measurements or arthritis scores, while the aforementioned *in vivo* measurements more closely paralleled inflammation scores. The increased sensitivity of the DAI to total histopathology, which is not only comprised of inflammation scores, but also pannus formation, cartilage damage, bone resorption, and periosteal bone formation scores, suggests that the DAI may provide more global information on functional capacity.

Rheumatoid Arthritis (RA) compounds with different therapeutic profiles have been described in the literature. The JAK inhibitor JTE-052, an anti-inflammatory compound similar to tofacitinib, and the calcineurin inhibitor tacrolimus, an immunosuppressants have been shown to improve spontaneous locomotion with partial inhibition of articular inflammation (Sasakawa et al., 2005; Tanimoto et al., 2015). In clinical trials, denosumab, a human monoclonal antibody that binds and inhibits RANKL, suppresses bone damage without affecting disease activity (Cohen et al., 2008). In the current study, the DAI identified slightly more etanercept (ENT)-treated rats compared to ankle joint measurements or arthritis scores. The two additional ENT-treated rats identified by the DAI scoring method were determined to have mild disease by histopathology (scores of 0.5 and 1.5). Etanercept, an inhibitor of the pro-inflammatory cytokine superfamily tumor necrosis factor (TNF), has been shown to be more effective in reducing ankle joint inflammation than other activity-dependent symptoms, such as pain (Chakravarthy et al., 2014).

We also demonstrate that the DAI complements standard methods and can be used to discern differential responses of novel or repurposed compounds, which may subsequently provide insights on its mechanism of action and potential therapeutic uses. For example, celecoxib showed greater disease attenuation as measured by ankle joint sizes, arthritis scores, and histopathology inflammation scores compared with total histopathology scores and the DAI. Celecoxib (generic name: Celebrex) is a cyclooxygenase-2 (COX-2)-selective non-steroidal anti-inflammatory drug (NSAID). The mechanism of action of celecoxib is to specifically block COX-2 enzymes that synthesize prostaglandins, subsequently reducing inflammation. As a current treatment for RA patients, NSAIDs are used to alleviate pain and stiffness (Emery et al., 1999; Simon et al., 1999). Since it does not slow down joint damage, it is normally used in-conjunction with other disease modifying anti-rheumatic drugs (DMARDs). Although further investigation is required, this disease profile provides an example of a compound that may have stronger anti-inflammatory and analgesic effects in comparison to attenuating longer-term joint damage.

In contrast, beclomethasone showed greater disease attenuation by the DAI compared with arthritis scores and histopathology. Beclomethasone is a synthetic glucocorticoid with anti-inflammatory and immune-modulating properties, which inhibits pro-inflammatory cytokine production. As a current treatment for RA patients, glucocorticoids have been shown to alleviate pain, joint swelling, and sudden flares of joint pain (Townsend and Saag, 2004). A study investigating glucocorticoid monotherapy also described improved function as measured by grip strength and improved radiographic scores, which incorporate measurements of joint space narrowing and bone erosions (van Everdingen et al., 2002). However, glucocorticoids are often used in-conjunction with other DMARDs as a “bridge therapy” due to the more rapid onset of its beneficial effects, but also due to its potential long-term adverse effects, including osteoporosis, cardiovascular and gastrointestinal side effects, hyperglycemia, and infection. This disease profile provides an example of a compound that may have stronger effects in improving short-term function and mobility in comparison to underlying inflammation and longer-term joint damage.

Lastly, phenanthridinone and ouabain octahydrate both showed certain degrees of disease attenuation as detected by the DAI, standard measurements, and histopathology. Phenanthridinone is an inhibitor of the nuclear enzyme poly(ADP-ribose) polymerase (PARP), which is implicated in the cellular response to oxidant-induced DNA damage. Previous studies have described the role of PARP inhibition in reducing inflammatory injury in rodent models of RA, streptozotocin-induced diabetes, and stroke (Rosado et al., 2013; Ahmad et al., 2014; Garcia and Conde, 2015). On the other hand, ouabain octahydrate, a sodium-potassium ATPase inhibitor, has not been previously tested in models of RA. Although its relationship with inflammation remains elusive, a previous study has shown that treatment with digoxin, a sodium-potassium ATPase inhibitor, impaired the Chikungunya virus (CHIKV), a mosquito-borne arthritogenic alphavirus that results in debilitating polyarthralgia and arthritis in infected individuals (Ashbrook et al., 2016). Further investigation is required to explore the therapeutic effects of PARP inhibitors and sodium-potassium ATPase inhibitors in RA; however, these preliminary results from our phenotypic screen suggest compounds that can potentially increase function and mobility, decrease inflammation, and improve longer-term joint damage.

The current study possesses some limitations. Although joint destruction has begun in CIA Vehicle rats by study termination (17 days post-induction), joint destruction did not reach full severity (see histopathology for CIA Vehicle rats in **Figure 6C**, Supplementary Figure 1C, and Table 1), thereby limiting the range of pathology by which to assess the full breadth of the DAI’s utility. In future studies, it will be interesting to see how well the DAI correlates with histopathology in later disease stages when compared against standard *in vivo* methods, as well as how the DAI profiles clinically effective compounds with different mechanisms of action. There are also several caveats of using the DAI to assess RA disease progression. Unlike arthritis scores, ankle joint measurements, or imaging modalities, the DAI does not quantify morphological severity or provide spatiotemporal information of disease severity. Due to the nature of ankle joint measurements, arthritis scoring and imaging techniques, experimenters are knowledgeable not only of which limbs are affected, but also the severity for each limb.

As an indirect readout of disease severity, the DAI is activity-dependent and may therefore be affected by a number of factors, including housing conditions, pain, fatigue, and mood disorders that are present in RA patients (Sasakawa et al., 2005; Pham et al., 2010; Martin et al., 2015; Tanimoto et al., 2015; Sturgeon et al., 2016). In the clinic, pain normally presents as the first reported symptom by RA patients, can be present without apparent swelling and synovitis, and affects overall quality of life, including mobility (Kidd et al., 2007; Lee et al., 2014). A number of studies have investigated the relationship between spontaneous activity and pain behavior in rodent RA models (Millecamps et al., 2005; Inglis et al., 2007). Previous studies have also shown the association between chronic pain driven by central nervous system (CNS) sensitization and neuroimmune mechanisms; moreover, the onset of pain hypersensitivity occurred earlier than hind paw and joint swelling in RA rodent models (Nieto et al., 2015, 2016). Therefore, for future studies, it will be interesting to investigate the relationship between pain and the DAI using reference compounds that have been shown to alleviate both pain and RA inflammation symptoms (i.e., celecoxib), compounds that attenuate the DAI to a greater degree than swelling and inflammation (i.e., beclomethasone), compounds that are used to generally alleviate pain (i.e., morphine), or compounds that may not have anti-inflammatory effects, but may affect activity measurements (i.e., anti-psychotic drugs). Furthermore, it will be interesting to investigate whether compounds that lead to earlier improvements in the DAI may be related to pain relief and whether compounds that lead to longer-lasting effects in the DAI may be related to long-term improvements in functionality and pathology. Future studies will continue to validate the DAI using reference compounds that robustly attenuate inflammation. A compound that improves the DAI without generating obvious improvements in inflammation and vice versa require further assessments and endpoint histopathology analysis in order to better investigate the therapeutic efficacy of the compound.

Finally, although rank ordering of compounds based on their response to various standard measurements in rodent models cannot completely predict therapeutic response in humans, a better understanding of the different disease profiles generated by these compounds can inform researchers and clinicians on appropriate course of treatment, including combination therapies, especially since arthritis symptoms evolve over time. Further investigating the disease profiles of compounds in other established RA models can provide more insight.

A challenge in RA preclinical research is demonstrating translation to the clinic. To address this challenge, scientists and clinicians are striving to develop sensitive methods that determine improvement not only of specific disease symptoms, but also of overall functional capacity (Welsing et al., 2001; Pincus et al., 2004; Yazici et al., 2004; Bombardier et al., 2012). While preclinical RA studies assess drug efficacy by relying on clinical scoring, joint swelling, and imaging modalities, clinical trials frequently index disease severity and therapeutic efficacy using changes in physician’s and patient’s reports of disease activity, functional ability, and pain (Felson et al., 1993). In RA animal models, activity and gait measurements have been shown to better correlate with joint damage compared to arthritis scores later in disease (Hayer et al., 2016). In RA patients, functional assessments, such as the presence of morning stiffness, reflect disability and pain, factors dramatically affecting quality of life in RA patients. These assessments have been shown to be comparable to disease activity scoring methods (Yazici et al., 2004). Similarly, to reduce the subjective nature of these assessments of physical activity in RA patients, clinicians and researchers are outfitting subjects with movement tracking devices, such as the Fitbit^®^ (Chiauzzi et al., 2015). Retrospective studies demonstrate the relationship between functional disability and mortality and hospitalization not only in RA, but also in other conditions (Pincus et al., 2004). Our results not only demonstrate that the DAI generally correlates well with standard methods for measuring disease severity in a preclinical RA model, but also that the DAI provides insight on functional changes during disease. By complementing standard methods, the DAI can be a physiologically relevant readout, providing researchers a parallel measurement of disease progression, thereby conceivably increasing translation to the clinical setting.

One of the largest challenges of drug discovery is the ability to rapidly identify compounds using *in vivo* disease models through phenotypic screens. *In vivo* animal models can be labor-intensive and can require post-mortem histopathology analysis, which may take several weeks to generate results (Perrin, 2014; Sakic et al., 2015). Screens for RA drugs are similarly laborious, due to the limitations of current methods, such as the need to have the same trained technician perform all conventional measurements. By combining computational and physiological approaches, we have developed an automated and systematic phenotyping platform to screen compounds in parallel in animal disease models, such as the CIA rodent model of RA, thus enabling researchers to make more efficient and informed evaluations for future experiments. This platform facilitates objective and reproducible metrics that can detect disease signals and complements standard methods. We believe that these tools will contribute to rapid, reproducible, and meaningful drug discovery approaches for preclinical research.

## Author Contributions

ML, DF, KH, and LS performed data analysis. PC managed and participated in the execution of studies. BL and PL developed the SMarTR^TM^ computational drug discovery engine. ML, DF, KH, PC, JB-L, TR, and LS contributed toward the development of the Vium Digital Platform. ML and LS wrote and edited the manuscript.

## Conflict of Interest Statement

ML, DF, KH, PC, JB-L, TR, and LS work and consult for Vium, Inc., who developed the Vium Digital Platform and the Digital Arthritis Index. BL and PL work for Capella Biosciences, Inc, who developed the SMarTR^TM^ computational drug discovery engine.
