# Supplementary material for: Development of the Digital Arthritis Index, a Novel Metric to Measure Disease Parameters in a Rat Model of Rheumatoid Arthritis
Source: Front Pharmacol. 2017 Nov 14;8:818. doi: 10.3389/fphar.2017.00818 (PMC5694443; doi:10.3389/fphar.2017.00818)
Supplement: Supplementary file 1 [file Table_1.pdf]

Supplementary Table 1. Breakdown of histopathology score in three independent experiments

	Experiment 1		Experiment 2		Experiment 3	
	CIA	Control	CIA	Control	CIA	Control
Inflammation	3.83 ± 1.14	0.11 ± 0.18	4.32 ± 1.16	NA	4.75 ± 0.37	NA
Pannus	0.70 ± 1.14	NA	0.79 ± 0.39	NA	1.44 ± 0.54	NA
Cartilage Damage	1.55 ± 0.77	0.02 ± 0.08	2.29 ± 0.81	NA	2.48 ± 0.69	NA
Bone Resorption	0.68 ± 0.41	NA	0.79 ± 0.39	NA	1.44 ± 0.54	NA
Periosteal Bone Formation	0.35 ± 0.34	NA	0.57 ± 0.28	NA	0.70 ± 0.51	NA
Total Score	7.10 ± 2.87	0.14 ± 0.25	8.75 ± 2.61	NA	10.81 ± 2.4	NA

Values represent means ± std. For Experiment 1, n=9/group. For Experiment 2, n=7/group. For Experiment 3, n=4 for CIA rats and n=16 for Control rats.
