# Supplementary material for: Development of the Digital Arthritis Index, a Novel Metric to Measure Disease Parameters in a Rat Model of Rheumatoid Arthritis
Source: Front Pharmacol. 2017 Nov 14;8:818. doi: 10.3389/fphar.2017.00818 (PMC5694443; doi:10.3389/fphar.2017.00818)
Supplement: Supplementary file 2 [file Image_1.pdf]

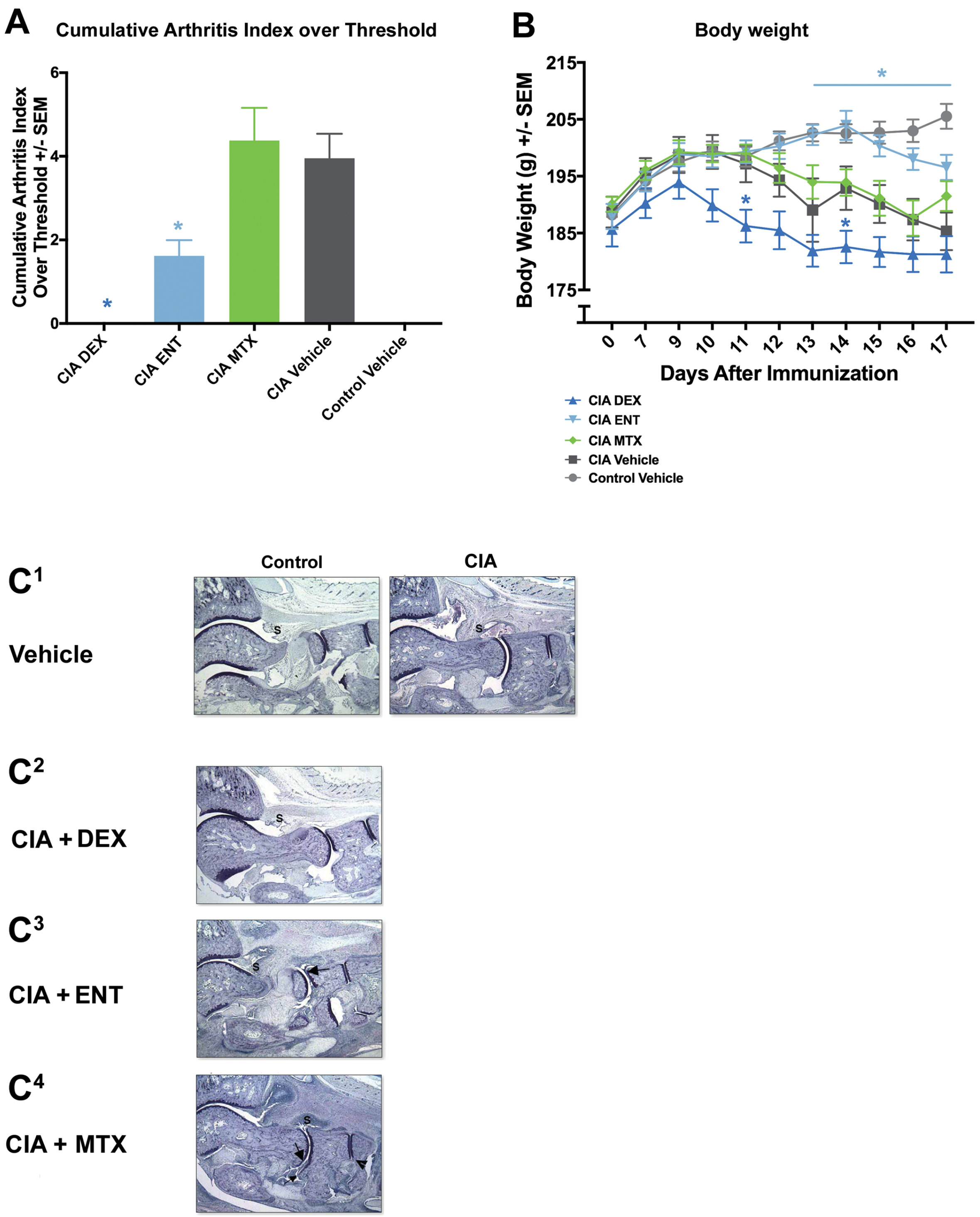

Supplementary Figure 1

Supplementary Figure 1. Additional disease parameters of standard of care (SOC)-treated rats. (A) Cumulative Arthritis Index (DAI) over threshold detects therapeutic efficacy of etanercept (ENT) and dexamethasone (DEX)-treated rats. (**B**) Body weight profiles over time reveal improved weights for ENT-treated, but not DEX-treated rats. **(C)** Representative ankle joint histopathology images for vehicle-treated controls, as well as DEX, ENT, and MTX-treated rats. **(C^{1})** Left panel: Vehicle-treated control rat showed a normal synovium (S). Right panel: Vehicle-treated, CIA-induced rat showed severe inflammation in synovium (S), minimal cartilage damage (large arrow), pannus, and bone resorption (not visible in 16x magnification). **(C^{2})** DEX-treated rat showed a normal synovium (S). **(C^{3})** ENT-treated rat showed mild inflammation (S), with very minimal cartilage damage and pannus (not visible in 16x magnification). **(C^{4})** MTX-treated rat showed severe inflammation (S), mild cartilage damage (large arrow), minimal pannus (small arrow) and bone resorption (arrowhead), as well as minimal periosteal bone formation (not visible in 16x magnification). S = synovium (indicating area of possible inflammation), large arrow = cartilage damage, small arrow = pannus, arrowhead = bone resorption, P = periosteal bone formation. Note that pannus, bone resorption, and periosteal bone formation are not always evident in 16x magnification). *$P< 0.05$ from CIA Vehicle. Error bars are SEM. n=9/group.
