# Supplementary material for: Development of the Digital Arthritis Index, a Novel Metric to Measure Disease Parameters in a Rat Model of Rheumatoid Arthritis
Source: Front Pharmacol. 2017 Nov 14;8:818. doi: 10.3389/fphar.2017.00818 (PMC5694443; doi:10.3389/fphar.2017.00818)
Supplement: Supplementary file 3 [file Image_2.pdf]

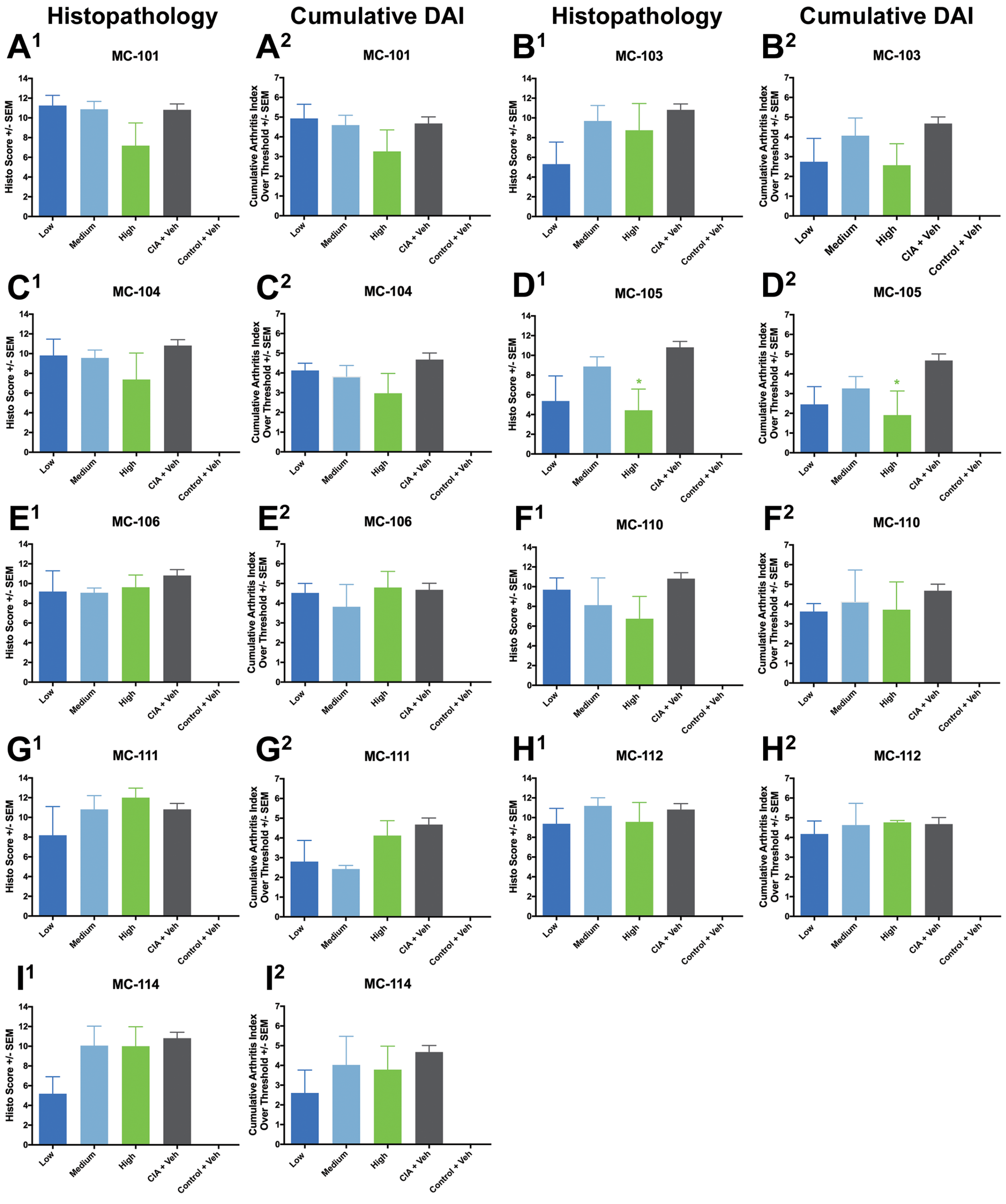

Supplementary Figure 2

Supplementary Figure 2. Histopathology scores and cumulative arthritis index (DAI) over threshold for nine repurposed compounds. Histopathology scores **(A^1-I^1)** and the cumulative arthritis index (DAI) over threshold **(A^2-I^2)** for each compound separated for low, medium, and high doses. Although specific doses of compounds showed trends towards improvement, only the high dose of MC-105 was significantly decreased compared to CIA Vehicle rats (CIA + Veh) (*$P \leq 0.05$). CIA + Veh rats showed a significantly elevated histopathology score and cumulative arthritis index compared to Controls (Control + Veh) ($P \leq 0.05$). Error bars are SEM. For each dosing group, n=4. For CIA Vehicle rats, n=4. For Control Vehicle rats, n=16.
