# Supplementary material for: Development of the Digital Arthritis Index, a Novel Metric to Measure Disease Parameters in a Rat Model of Rheumatoid Arthritis
Source: Front Pharmacol. 2017 Nov 14;8:818. doi: 10.3389/fphar.2017.00818 (PMC5694443; doi:10.3389/fphar.2017.00818)
Supplement: Supplementary file 4 [file Image_3.pdf]

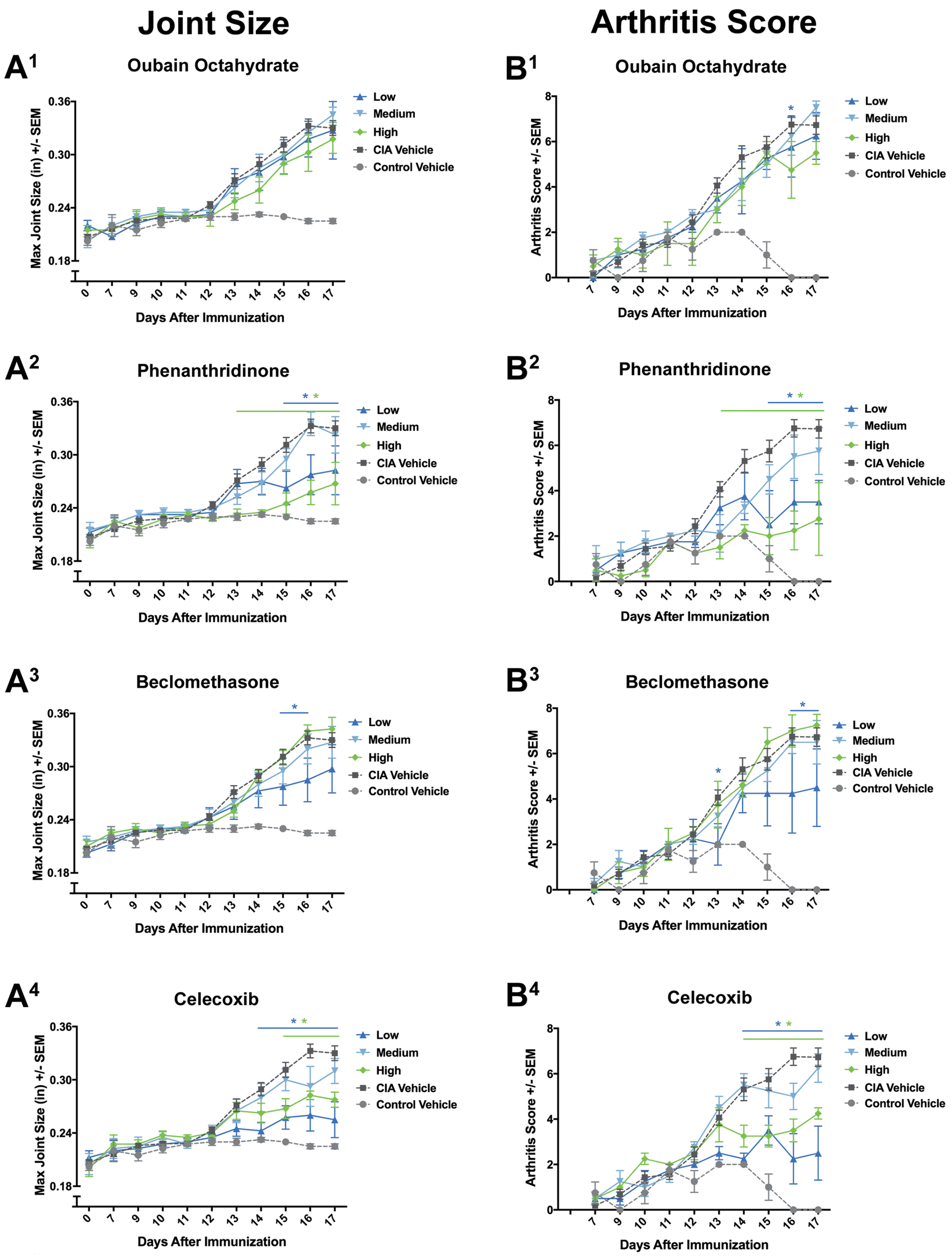

Supplementary Figure 3

Supplementary Figure 3. Standard *in-life* disease parameters of a subset of treated rats. Dose-dependent improvement was also detected by ankle joint size measurements and arthritis scores for rats with treated with ouabain octahydrate **(A^1, B^1)**, phenanthridinone **(A^2, B^2)**, beclomethasone **(A^3, B^3)**, and celecoxib **(A^4, B^4)**. *$P<0.05$ from CIA Vehicle. Error bars are SEM. For each dosing group, n=4. For CIA Vehicle rats, n=4. For Control Vehicle rats, n=16.
